# Stimulus‐Responsive Gas Marbles as an Amphibious Carrier for Gaseous Materials

**DOI:** 10.1002/advs.202404728

**Published:** 2024-06-25

**Authors:** Takanori Yasui, Anne‐Laure Fameau, Hyoungwon Park, Thu Thao Pham, Sabrina Pechmann, Silke Christiansen, Shin‐ichi Yusa, Tomoyasu Hirai, Yoshinobu Nakamura, Syuji Fujii

**Affiliations:** ^1^ Graduate School of Engineering Osaka Institute of Technology 5‐16‐1, Omiya, Asahi‐ku Osaka 535‐8585 Japan; ^2^ Université Lille CNRS INRAE Centrale Lille UMR 8207 – UMET – Unité Matériaux et Transformations Lille F‐59000 France; ^3^ Department for Correlative Microscopy and Materials Data Fraunhofer Institute for Ceramic Technologies and Systems (IKTS) 91301 Forchheim Germany; ^4^ Department of Applied Chemistry Graduate School of Engineering University of Hyogo 2167 Shosha Himeji Hyogo 671‐2280 Japan; ^5^ Institute for Nanotechnology and Correlative Microscopy gGmbH (INAM gGmbH) 91301 Forchheim Germany; ^6^ Fachbereich Physik Freie Universität Berlin (FU Berlin) 14195 Berlin Germany; ^7^ Department of Applied Chemistry Faculty of Engineering Osaka Institute of Technology 5‐16‐1, Omiya, Asahi‐ku Osaka 535‐8585 Japan; ^8^ Nanomaterials Microdevices Research Center Osaka Institute of Technology 5‐16‐1 Omiya, Asahi‐ku Osaka 535‐8585 Japan

**Keywords:** adsorption, amphibious, carrier, gas marbles, interface, stimulus‐responsive

## Abstract

Gas marbles are a new family of particle‐stabilized soft dispersed system with a soap bubble‐like air‐in‐water‐in‐air structure. Herein, stimulus‐responsive character is successfully introduced to a gas marble system for the first time using polymer particles carrying a poly(tertiary amine methacrylate) (p*K*_a_ ≈7) steric stabilizer on their surfaces as a particulate stabilizer. The gas marbles exhibited long‐term stability when transferred onto the planar surface of liquid water, provided that the solution pH of the subphase is basic and neutral. In contrast, the use of acidic solutions led to immediate disintegration of the gas marbles, resulting in release of the inner gas. The critical minimum solution pH required for long‐term gas marble stability correlates closely with the known p*K*_a_ value for the poly(tertiary amine methacrylate) stabilizer. It also demonstrates amphibious motions of the gas marbles.

## Graphical abstract

It successfully produced stimulus‐responsive “gas marbles” using sterically stabilized polystyrene particles. The pH‐responsive steric stabilizer chains on the particle surface enabled on‐demand disruption of the gas marbles trough pH control, leading to release of the inner gas. The gas marbles exhibited amphibious motion, indicating their potential for applications in the encapsulation, delivery, and release of gaseous materials.

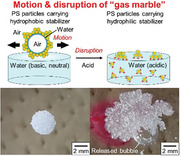

## 1 Introduction

Over the past few decades, the adsorption of solid particles at the oil‐water and air‐water interfaces has garnered growing interest.^[^ ^1^, ^2^ ^]^ New concepts and materials have emerged in this developing soft matter research field, such as Ramsden/Pickering emulsions,^[^ ^3^, ^4^, ^5^, ^6^ ^]^ colloidosomes,^[^ ^7^, ^8^ ^]^ liquid marbles,^[^ ^9^, ^10^, ^11^, ^12^, ^13^, ^14^ ^]^ dry liquids,^[^ ^15^ ^]^ armored bubbles,^[^ ^16^, ^17^, ^18^ ^]^ and foam marbles,^[^ ^19^ ^]^ with various promising applications including miniature reactors for chemical and biological reactions, carriers of materials in microfluidics, food, and bio‐related technologies. The field of soft materials that exhibit responses to external stimuli stands at the forefront of scientific exploration and is a fascinating and rapidly evolving domain with a plethora of unexplored commercial applications.^[^ ^20^, ^21^, ^22^, ^23^, ^24^ ^]^ Although researchers face compelling challenges within this dynamic field, the potential exists for significant advances in the intricate realms of the conceptualization, synthesis, and engineering of stimuli‐responsive materials, particularly those based on particles adsorbed at interfaces. A new family of particle‐stabilized dispersed systems called ʻgas marbles (GMs)ʼ has appeared in recent literature.^[^ ^25^, ^26^, ^27^ ^]^ GMs have an air‐in‐water‐in‐air structure resembling soap bubbles. In the first studies on GMs, commercially available particles with unknown surface chemistry were utilized so that the particle properties that can stabilize GMs are yet under a veil. Moreover, the structure of GMs has not yet been fully evaluated and, to our best knowledge, the design of stimulus‐responsive GMs has not been demonstrated prior to this report.

**Figure 1:**
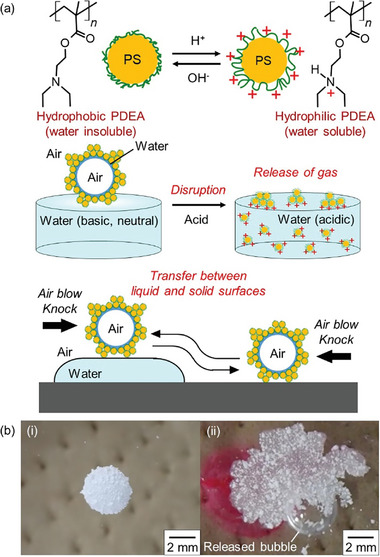
a) pH‐Responsive gas marble (GM) stabilized with polystyrene (PS) particles carrying a poly[2‐(diethylamino)ethyl methacrylate] (PDEA) steric colloidal stabilizer (PDEA‐PS particles). Amphibious motion of the GM.; b) Disruption of GM (bubble volume, 20 µL), followed by release of gas, on the planar air‐water interface by addition of a HCl aqueous solution (dyed using rhodamine B); Images were taken (i) before and (ii) after the addition of the HCl aqueous solution.

Herein, we have described the synthesis of polystyrene (PS) particles carrying a pH‐responsive poly[2‐(diethylamino)ethyl methacrylate] (PDEA) steric colloidal stabilizer on their surfaces (PDEA‐PS particles) and evaluated their ability as a GM stabilizer to produce a stimulus‐responsive ʻGMsʼ system (**Figure** **1**a). In theory, synthetic polymer particles are a particularly attractive stabilizer for GMs since their surface chemistries can be readily designed. Researchers have studied a series of polymer particles as stabilizers for emulsions,^[^ ^28^ ^]^ foams,^[^ ^29^ ^]^ liquid marbles,^[^ ^30^ ^]^ and foam marbles.^[^ ^19^ ^]^ The most crucial element in defining the interfacial properties of the polymer particles is, in each instance, their surface chemistry. Due to the poly(tertiary amine methacrylate)‐based steric stabilizer on the particle surface, it is possible to create stable GMs, which can then be disrupted, leading to the release of the gas, on‐demand at low pH. Furthermore, we have demonstrated the motions of GMs both on planar air‐water and air‐solid interfaces. The developed system could be applied for encapsulation/delivery/release of gaseous materials.

## 2 Results and Discussion

PDEA is a pH‐responsive polybase with a p*K*_a_ value of ≈7.0.^[^ ^31^, ^32^, ^33^ ^]^ PDEA exhibits a hydrophobic character and precipitates at pH 8 or above in aqueous media in a neutral non‐protonated form. On the other hand, PDEA is strongly hydrophilic and water soluble below pH 6 since the protonated PDEA is in a polyelectrolytic form. Here, in order to synthesize PDEA‐PS particles, the PDEA homopolymer, which was synthesized via solution polymerization, was employed as a steric colloidal stabilizer. The number‐average molecular weight and the polydispersity index (*M*_w_/*M*_n_) of the PDEA homopolymer were 9 600 g mol^−1^ and 1.26, respectively, as determined by gel permeation chromatography. Dispersion polymerization of styrene conducted in isopropanol using the PDEA stabilizer in a ʻone‐shotʼ batch manner led to the successful synthesis of colloidally stable PDEA‐PS particles with no coagulum (Figure S1a, Supporting Information). Scanning electron microscopy (SEM) observations of dried PDEA‐PS particles obtained after centrifugal purification confirmed their spherical morphology, from which the number average particle diameter (*D*_n_) was estimated to be 1.58 ± 0.03 µm (Figure S1b, Supporting Information). Elemental microanalysis investigations revealed that the PDEA loading percentage in the PDEA‐PS particles amounted to 1.3 wt.%, which was also supported by ^1^H NMR studies (Figure S2, Supporting Information). Since PDEA is soluble in isopropanol but not in PS, it is anticipated that PDEA predominantly resides at the surface of the PDEA‐PS particles. This assertion finds support in X‐ray photoelectron spectroscopy studies (Figure S3, Supporting Information), suggesting a surface coverage of the PDEA stabilizer on the PS particles of 18.7%.

Assuming that all PDEA chains are situated at the surface of the PS particles (Figures S1c–S4, Supporting Information) and considering the *D_n_* of the PS particles as 1.58 ± 0.03 µm, the surface concentration of PDEA was calculated to be 3.7 mg m^−2^, using the equation *As* = *3/ρR* (where *As* is the surface area per unit mass, *ρ* is the density of PS (1.06 g.cm^−3^), and *R* is the radius of the particles) (Supporting Information). This value is comparable to those reported for PS particles prepared by alcoholic dispersion polymerization using poly(*N*‐vinyl pyrrolidone) (PNVP) stabilizer (4.5 mg.m^−2^).^[^ ^34^ ^]^ The contact angles of the aqueous solutions of HCl (pH 3), deionized water, and NaOH (pH 10) were measured to be 55 ± 1°, 81 ± 1°, and 89 ± 1°, respectively, indicating the pH‐dependent wetting behavior of the PDEA‐PS particles.

**Figure 2:**
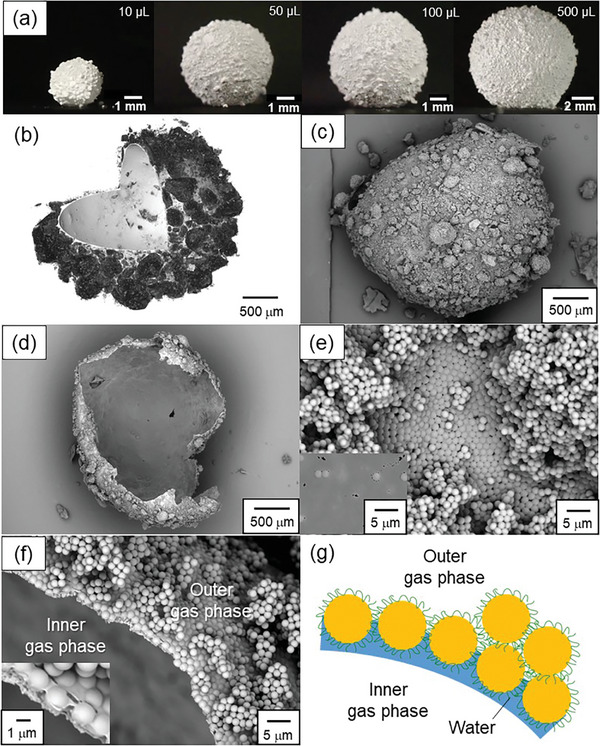
a) Optical photographs of GMs stabilized by PDEA‐PS particles prepared with various bubble volumes; b) X‐ray microscopy image of GM fabricated using a water/glycerol (1/1, w/w) mixture; c–f) SEM images of GM (bubble volume, 10 µL) after ethyl cyanoacrylate vapor treatment, followed by the evaporation of water: c,e) Outer and d) inner surfaces; and f) cross‐sectional images of GM; The GM was crushed using a plastic spatula; Inset in e) shows inner surface of GM; Inset in f) is a magnified image; g) Illustration of GM cross‐section.

Individual GMs were fabricated as follows (Figure S5, Movie S1, Supporting Information): Air bubbles with controlled volumes were injected below the planar air‐water interface covered by the PDEA‐PS particle raft using a syringe, resulting in the formation of bubbles whose upper surface is covered by the particle raft. The bubble was then pushed toward the surrounding particle raft using a dispensing spoon and was rolled over the particles so that they covered the entire bubble surface. The PDEA‐PS particles autonomously coat the aqueous bubble and render it non‐wetting. The resulting GMs remained intact even after transfer onto a hydrophilic glass slide (**Figure** **2**a) and could move by application of physical forces, such as knocking or air blowing (Movies S2 and S3, Supporting Information). The gas exchange could occur between the inner and outer air, because of the solubility of air in the water phase. However, insoluble gas could be used in small amounts to stop the gas exchange between the inside of the GMs and its environment, as already demonstrated for aqueous liquid foams.^[^ ^35^ ^]^ In control experiments, no GMs could be formed using sterically‐stabilized PS particles synthesized using intrinsically hydrophilic stabilizers such as PNVP; such PS particles started to disperse into the aqueous medium just after placement on the planar air‐water interface. These observations confirm the essential role played by the PDEA stabilizer in promoting GM formation.

**Figure 3:**
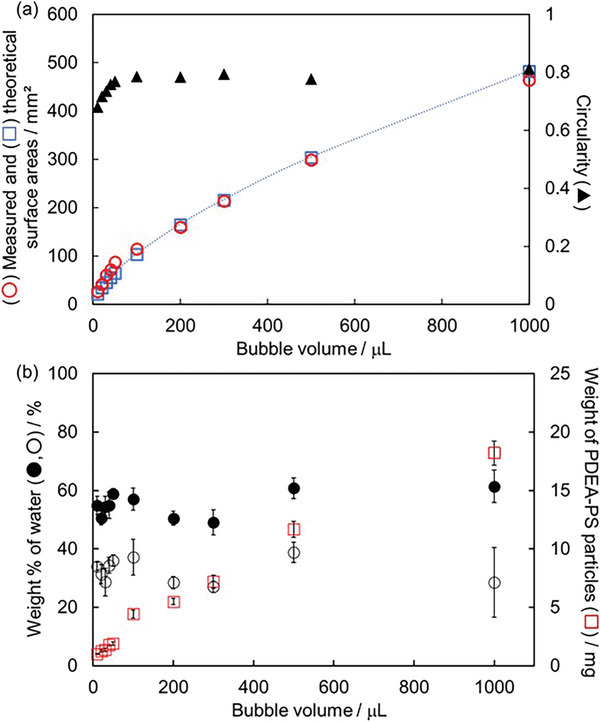
a) Comparison of (○) measured surface area of GM, (□) theoretical surface area of gas bubble, and (▲) circularity of GM. Measured surface areas were calculated using the radius determined from the average of the height and width of GM. Theoretical surface areas were calculated based on theoretical formula ($S=\sqrt[{3}]{36\pi V^{2}}$) assuming spherical shape of GM (solid line). *S* is surface area and *V* is bubble volume; b) Weight % of water (●,○) and weight of PDEA‐PS particles (□) adsorbed to the GM depending on bubble volume in GM. (●) Weight % of water just after fabrication of GM, and (○) weight % of water when the GM was disrupted at humidity of 71%. Drying time: 24 h.

**Figure** **3**a shows how the surface area of GMs varied with the volume of the bubbles used for their preparation. The theoretical surface area calculated under the assumption that the GMs have a spherical shape accorded well with the results determined experimentally. The circularity of the GMs were almost constant (0.81–0.84) independent of gas volume (50–1000 µL). This is strikingly different from the results obtained for liquid marbles, i.e., water droplets coated by PDEA‐PS particles, in that the circularity starts to decrease at and above the capillary length (volume, ≈10 µL) due to the effect of gravity:^[^ ^36^ ^]^ the circularity of 1000 µL liquid marble was 0.62 (Figure S6, Supporting Information). The weights of the PDEA‐PS particles adsorbed to the GMs depending on the gas volume showed the same trend as the surface area (Figure 3b). These results indicated that the thicknesses of the PDEA‐PS particle coatings were almost the same independent of the gas volume. Interestingly, the weight percentages of water had almost the same values (49–61 wt.%), suggesting the same thickness of the water layer was formed for the GMs (26–93 µm).

GMs clearly have significant surface roughness, indicating the existence of aggregates of the PDEA‐PS particles rather than just a monolayer on the GM surfaces (Figure 2a). X‐ray microscopy studies of GM (water/glycerol, 1/1, w/w; gas volume, ≈10 µL) confirmed the existence of a few tens to a few hundreds micrometer‐sized aggregates of the particles adsorbed on the outer surface of the liquid thin layer while no/few aggregates could be observed on the inner side of the liquid thin layer (Figures 2b; Figure S7 and Movie S4, Supporting Information). In order to investigate the structure of this particle coating layer in more detail, we treated the GM with ethyl cyanoacrylate (ECA) vapor to trap the particles at the air‐water interface, followed by SEM observations^[^ ^37^ ^]^ (Figure 2c–f). After ECA vapor treatment, the poly(ethyl cyanoacrylate) (PECA) film was formed at the air‐water interface via anionic polymerization initiated by water catalyst to form capsules, which did not rupture even after complete evaporation of water. SEM imaging of the outer surface of the GMs confirmed the dual morphology of PDEA‐PS particle aggregates and particle arrays (Figure 2e). On the other hand, the smooth PECA film with a few particles trapped was observed in the inner surface (Figure 2d, inset of 2e). Cross‐sectional SEM studies clearly showed that the PDEA‐PS particle aggregates attached to the outer surfaces of GMs and the PDEA‐PS particle array was trapped in the PECA film as a monolayer (Figures 2f,g; Figure S8, Supporting Information). This morphology was different from that proposed previously, where particles were bridged by a water layer.^[^ ^26^ ^]^ The particle aggregates were found in the PDEA‐PS particle raft on the planar air‐water interface rather than particle array monolayer because particle‐particle interaction dominates over gravity for a few micrometer‐sized polymer particles, and these aggregates cover the GMs (Figure S9, Supporting Information). A particle array monolayer could be formed during the preparation of GMs by rolling, which caused the partial collapse of the aggregates into independent PDEA‐PS particles.

**Figure 4:**
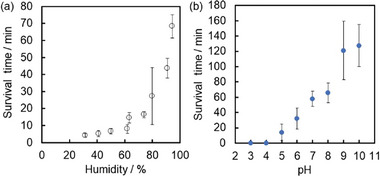
a) Relationship between humidity and survival time of GMs on hydrophobic solid surface (bubble volume, 20 µL). The humidity was controlled using saturated aqueous solutions of various salts; b) pH‐Dependent stability of GMs (bubble volume, 20 µL) placed on planar air‐water interface with various pH values at 21 °C and 80 RH%.

Sudden disruption of the GMs on the hydrophobic solid substrate occurred in a time scale of 10–20 min at 63%RH due to water evaporation, followed by rupture of the water layer. The weight percentages of water in the GMs at disruption were almost the same (27–39 wt.%) (Figure 3b), which was independent of the size of the GMs. These results supported our observations that the water layer thickness for GMs of various sizes was the same, and the thickness of the water layer when the GMs collapsed was estimated to be 9–36 µm. Stability of the GMs could be enhanced to 70 min with an increase of humidity up to 95%RH due to the reduction of water evaporation (**Figure** **4**a). The addition of an electrolyte in the aqueous phase could also extend the stability time (over 2 days in the case of a saturated aqueous solution of NaCl and MgCl_2_), since the involatile electrolyte reduced the rate of evaporation. Using an aqueous solution of glycerol (50 wt.%) as the inner liquid realized extremely long stability (> 6 months).^[^ ^27^ ^]^ Therefore, the stability of GMs can be enhanced from a few tens of minutes to months by modifying the properties of the thin water layer by adding electrolytes or glycerol. The GMs could also collapse by the application of mechanical stress to release the inner gas (Figure S10 and Movie S5, Supporting Information).

Noteworthily, the GMs were stable even after transfer onto a planar surface of liquid water and could move by knocking, air blowing or creation of a vertical deflection around the GM (Movies S6–S8, Supporting Information). Remarkably, the GMs can move back and forth between air‐water and air‐solid interfaces (Figure 1a; Movie S9, Supporting Information). The effect of the pH of the underlying water on the stability of individual GMs placed at the air‐aqueous solution interface was investigated (Figure 4b). The GMs exhibited long‐term stability (> 1 h) on the planar surface of liquid water, provided that the subphase had a pH ≥ 8. In contrast, the GMs placed on acidic solutions (pH <5) showed catastrophic destruction (<10 min), with dispersal of the PDEA‐PS particles in/on the aqueous solution. Clearly, the critical minimum solution pH required for long‐term stability of GMs correlates closely with the known p*K*_a_ value of 7.0 for the PDEA stabilizer chains.

It was thus observed that the PDEA‐PS particles confer the pH‐responsive behavior on the GMs (Figure 1b). The GM placed on the planar air‐water interface disintegrated within 1 s after addition of an aqueous solution of HCl to the bulk aqueous subphase (the solution pH decreased from 6.8 ± 0.1 to 3.0 ± 0.3). The disruption resulted in the release of the encapsulated bubble (Movie S10, Supporting Information). This pH‐induced disruption could also be realized by UV light irradiation on the GM floating at the planar air‐water interface containing a photo‐acid generator, leading to a decrease in pH upon UV light irradiation (Movie S11, Supporting Information): the GMs collapsed 9.6 ± 3.3 s after light irradiation. Without light irradiation, the GMs were stable for 6.2 ± 0.8 min. In control experiments, the same‐sized GMs remained stable for 77 ± 15 and 110 ± 8 min after the addition of deionized water and an aqueous solution of NaOH, respectively, under corresponding conditions. The disruption of the GMs by pH change could occur in a cascade manner: pH change of the water phase induced an increase in the wettability of the PDEA‐PS particles to aqueous medium upon protonation at nanometer scale, followed by detachment of the particles from the air‐water interface at micrometer scale, leading to its rapid disintegration and release of the inner gas at millimeter scale. Thus, GMs were seen to be multiscale systems, and their stability and responsiveness to external pH stimuli arise from a combination of these scales.

## 3 Conclusion

In summary, sterically‐stabilized polymer particles can be employed for the preparation of GMs. The fabrication of GMs whose stability can be controlled in a well‐defined manner by external stimulus could be accomplished by the insertion of stimulus‐responsive materials into the GM systems. While hydrophilicity‐hydrophobicity balance is one of vital factors in the formation and stability of GMs, other factors such as particle size, shape, concentration, and the properties of the liquid film should play significant roles. By optimizing these parameters, the fabrication strategy for GMs can be adapted to use a wide range of solid particles, enhancing their versatility. This study shows both the control of the amphibious motion and the controlled release of substances from the GM by applying external stimuli within the system using GMs as the carrier. This suggests potential applications in the encapsulation, delivery, and release of gaseous materials. For example, these GMs could be exploited as gas storage materials. By designing the film composition and inhibiting the gas permittivity, valuable or polluted gases can be encapsulated inside the bubbles with insignificant gas diffusion/exchange with the environment. They could be also used to create composite materials with embedded gas pockets, potentially leading to new materials with unique properties such as lightweight or enhanced thermal insulation. The future challenge is the fabrication of GMs on a large scale, which broadens their applications. Several approaches can be considered such as developing robotic systems that can automate the process of creating GMs or adapting aeration techniques commonly used in aquaculture and food industries.

## 4 Experimental Section

### Materials

Styrene, *α*,*α*’‐azobisisobutyronitrile (AIBN), isopropanol (IPA, 99%), hydrochloric acid (HCl, 0.5 m aqueous solution), aluminum oxide (activated, basic, Brockmann 1, standard grade, ≈150 mesh, pore diameter 58 Å), glycerol (>99.5%) and sodium hydroxide (NaOH, >98.0%) were purchased from Sigma–Aldrich. Ethyl 2‐cyanoacrylate (ECA, Aron Alpha Extra Sokkotayoto) was purchased from Toagosei Co. 2‐(Diethylamino)ethyl methacrylate (DEA, >98.5%), diphenyliodonium nitrate (>98.0%), Rhodamine B (>95.0%) was purchased from Tokyo Chemical Industry Co., Ltd. Potassium carbonate (K_2_CO_3_, >99.5%), magnesium chloride (MgCl_2_, >97.0%), potassium chloride (KCl, >99.0%) and sodium bromide (NaBr, >99.0%) were purchased from FujiFilm Wako Pure Chemical Co. Potassium sulfate (K_2_SO_4_, >99.0%) was purchased from Kanto Chemical Co., Inc. Styrene and DEA were treated with the basic alumina to remove the inhibitor and then stored at 0 °C prior to use. Distilled water for solution preparation was first ion exchanged to a resistance of 18.2 MΩ·cm and then distilled (Advantec MFS RFD240NA: GA25A‐0715).

### Preparation of PDEA Homopolymer

The PDEA homopolymer was synthesized by solution polymerization of DEA in IPA medium. The initiator AIBN (0.4 g, 2.44 mmol) and IPA (400 mL) were stirred in a round‐bottomed 1 L flask equipped with a magnetic stirrer bar until dissolved completely and bubbled for 30 min with nitrogen gas to purge oxygen at room temperature. Under a stream of nitrogen and with constant stirring at 250 rpm, the monomer DEA (40 g, 216 mmol) which was purified by passage through an aluminum oxide column was injected to the flask to start the polymerization at 70 °C using a temperature controlled magnetic stirrer. After the polymerization for 24 h, the reaction solution was cooled to room temperature.

### Synthesis of PDEA‐PS Particles

The PDEA‐PS particles were synthesized by dispersion polymerization according to previous study.^[^ ^38^ ^]^ Briefly, the IPA solution of PDEA homopolymer prepared by solution polymerization (58.8 g, 11.36 wt.%) and IPA (534 mL) were mixed in a 1 L round‐bottomed flask and equipped with a magnetic stirrer bar, and bubbled for 30 min with nitrogen gas to purge oxygen at room temperature. Under a stream of nitrogen and with constant stirring at 250 rpm, a mixture of the monomer styrene (60.5 g, 0.58 mol), which was purified by passage through an aluminum oxide column, and the initiator AIBN (0.6 g, 3.65 mmol) were injected to the flask to start the polymerization at 70 °C using magnetic stirrer and thermostatic bath. After the polymerization for 24 h, IPA dispersion of PDEA‐PS particles was cooled to room temperature. The IPA dispersion of PDEA‐PS particles was purified by centrifugation/redispersion cycles with IPA (4 cycles; 3000 rpm, 1207 *g*, 15 min) and then deionized water (4 cycles; 4500 rpm, 2717 *g*, 15 min) using a centrifuge (Hitachi, CF16RXII type centrifuge with a Hitachi T15A 36 rotor).

### Characterization of PDEA‐PS Particles—Scanning Electron Microscopy (SEM)

The droplets of centrifugally purified dispersion of the PDEA‐PS particles were dried on an aluminium stub and sputter‐coated with gold (a few tens nm thickness) using an Au coater (SC‐701 Quick Coater, Elionix, Japan) in order to minimize sample‐charging problems. SEM studies were carried out using Hitachi High‐Tech Co., Ltd. TM4000 SEM operated at 15 kV. Number‐average diameter of the PDEA‐PS particles (*n* = 150) was determined from the SEM images. Dried GMs were also observed using SEM.

### X‐ray Photoelectron Spectroscopy (XPS)

For XPS analyses, the dried PDEA‐PS particles immobilized by rubbing on the indium surface were placed under reduced pressure by continuous operation of a diffusion pump just before the measurement. The indium substrates were arranged onto the sample holder with a conductive tape. XPS spectra were recorded with a JPS‐9030 XPS (JEOL Ltd., Japan) using Mg K*α* X‐rays (1253.6 eV) at 100 W (10 mA, 10 kV). Wide scans were carried out over a 1200–0 eV binding energy range with 1 eV steps; narrow scans were carried out with 0.2 eV steps. Spectra were referenced to the hydrocarbon component of the C_1s_ signal at 285 eV.

### Drying Method

Aqueous dispersion of PDEA‐PS particles (50 g) was placed in a 70 mL glass vial (Sogo Laboratory Glass Works Co., Ltd) and then was frozen at −18 °C overnight. The frozen dispersion was freeze dried at room temperature and < 87.6 Pa using a freeze dryer (FDU‐1200, Tokyo Rikakikai Co., Ltd.). The dried sample was broken into powder using a spatula.

### Digital Photography

A digital camera (Tough TG‐6, Olympus) was used to record photographic images of the millimeter‐sized GMs.

### X‐ray Microscopy (XRM) Imaging

XRM imaging scans were obtained by means of a Zeiss Xradia Versa 620 (Carl Zeiss X‐ray Microscopy Inc., Pleasanton, CA, USA), with a high framerate CMOS detector installed. A total 1401 X‐ray projection radiographs were recorded at different angles with a field of view value of 4.4 mm and they were reconstructed by proprietary software (Zeiss XMReconstructor, Carl Zeiss Xray Microscopy Inc., Pleasanton, CA, USA) with a filtered back‐projection algorithm. The resulting volumetric images had a 3D isotropic voxel size of 4.3 µm. The enhanced resolution was attained through the synergistic application of geometric magnification derived from the cone‐shaped X‐ray beam and the additional magnification provided by a 4× objective lens. The 4.3 µm voxel size scan was recorded with 1.8 s exposure and a framerate of 12 with a voltage of 30 kV and 2 W. Data visualization was done with the workflow‐based modular software XamFlow (Lucid Concepts AG, Zürich, Switzerland). As a sample, GM (water/glycerol, 1/1, w/w; gas volume, ≈10 µL) was used. Here, aqueous solution of glycerol was utilized to attenuate the evaporation of liquid to retain the 3D structure and size of the GM during the XRM imaging scans.^[^ ^27^ ^]^

### Interfacial Particle Trapping Method

Air‐water interfacial particle trapping on the surface of GM was conducted following the method established by Vogel et al.^[^ ^16^ ^]^ The GM just after preparation was placed in the petri dish. ECA monomer (1.0 g) was placed in the other petri dish on a hotplate (60 °C). Both petri dishes were placed in a closed container for 30 min. The monomer can evaporate and polymerize at the air‐water interface. The anionic polymerization of ECA is initiated at the interface upon contact with water, and the polycyanoacrylate is generated. The polymerization reaction of ECA is initiated by nucleophiles (e.g., water molecules). More monomer is supplied via the gas phase, the polymerization proceeds to eventually cover the air‐water interface, embedding particles at the air‐water interface in their equilibrium position.

### pH‐Dependent/Responsive Stability of GMs

GMs fabricated using a bubble volume of 20 µL were deposited onto the surface of various aqueous solutions ranging from pH 3 to 10. The characteristic time required for catastrophic destruction of each GM was determined. In addition, the pH‐responsive character of the GMs (bubble volume, 20 µL) was assessed as follows. Stable GMs deposited onto de‐ionized water at pH 6.8 were rapidly destroyed in situ by the careful addition of a few drops of concentrated HCl aqueous solution (0.5 m) using a pipette to lower the solution pH to around 3. pH‐Responsive behavior was also assessed by UV light irradiation (HLR100T‐2, Sen Lights Co., Ltd, 170 mW cm^−2^) to the GM floating on a planar air‐water interface containing a photo‐acid generator (diphenyliodonium nitrate, 50 mm). After UV light irradiation, pH decreased from 6.60 to 3.95. The surface tensions of aqueous solution of diphenyliodonium nitrate were 70.46 ± 0.08 and 70.07 ± 0.46 mN m^−1^ before and after the UV irradiation.

## Conflict of Interest

The authors declare no conflict of interest.

## Supporting information

Supporting Information

Supplemental Movie 1

Supplemental Movie 2

Supplemental Movie 3

Supplemental Movie 4

Supplemental Movie 5

Supplemental Movie 6

Supplemental Movie 7

Supplemental Movie 8

Supplemental Movie 9

Supplemental Movie 10

Supplemental Movie 11

## Data Availability

The data that support the findings of this study are available in the supplementary material of this article.
