# Supplementary material for: Stimulus‐Responsive Gas Marbles as an Amphibious Carrier for Gaseous Materials
Source: Adv Sci (Weinh). 2024 Jun 25;11(32):2404728. doi: 10.1002/advs.202404728 (PMC11348068; doi:10.1002/advs.202404728)
Supplement: Supplementary file 1 — Supporting Information [file ADVS-11-2404728-s012.pdf]

ADVANCED SCIENCE
Open Access

Supporting Information

for *Adv. Sci.*, DOI 10.1002/advs.202404728

Stimulus-Responsive Gas Marbles as an Amphibious Carrier for Gaseous Materials

*Takanori Yasui, Anne-Laure Fameau, Hyoungwon Park, Thu Thao Pham, Sabrina Pechmann, Silke Christiansen, Shin-ichi Yusa, Tomoyasu Hirai, Yoshinobu Nakamura and Syuji Fujii**

Supporting Information for *Advanced Science*

'Stimulus-Responsive Gas Marbles

as an Amphibious Carrier for Gaseous Materials'

Takanori Yasui[1], Anne-Laure Fameau[2], Hyoungwon Park[3], Thu Thao Pham[4], Sabrina Pechmann[3], Silke Christiansen[3,5,6], Shin-ichi Yusa[4], Tomoyasu Hirai[1,7], Yoshinobu Nakamura[1,7], Syuji Fujii[1,7]*

[1] Department of Applied Chemistry, Faculty of Engineering, Osaka Institute of Technology, 5-16-1 Omiya, Asahi-ku, Osaka 535-8585, Japan
[2] Université Lille, CNRS, INRAE, Centrale Lille, UMR 8207 - UMET - Unité Matériaux et Transformations, F-59000 Lille, France
[3] Department for Correlative Microscopy and Materials Data, Fraunhofer Institute for Ceramic Technologies and Systems (IKTS), 91301 Forchheim, Germany
[4] Department of Applied Chemistry, Graduate School of Engineering, University of Hyogo, 2167 Shosha, Himeji, Hyogo 671-2280, Japan
[5] Institute for Nanotechnology and Correlative Microscopy gGmbH (INAM gGmbH), 91301 Forchheim, Germany
[6] Fachbereich Physik, Freie Universität Berlin (FU Berlin), 14195 Berlin, Germany
[7] Nanomaterials Microdevices Research Center, Osaka Institute of Technology, 5-16-1 Omiya, Asahi-ku, Osaka 535-8585, Japan

* Author to whom correspondence should be addressed
(syuji.fujii@oit.ac.jp)

Number of pages in the Supporting Information: 20 (page S1 to page S20)

Number of Tables in the Supporting Information: 1 (Table S1)

Number of Figures in the Supporting Information: 10 (Figure S1 to Figure S10)

Number of Movies in the Supporting Information: 11 (Movie S1 to Movie S11)

Experimental Section

Characterization of poly[2-(diethylamino)ethyl methacrylate] (PDEA) homopolymer

Gel-permeation chromatography (GPC) measurement for PDEA was performed using a Jasco RI-2031 refractive index detector (Jasco, Tokyo, Japan) equipped with a CTO-10AS VP Shimadzu column working at 40 °C under a flow rate of 0.60 mL/min. A 0.3 M Na_2SO_4 aqueous solution containing a 0.5 M acetic acid was used as eluent and delivered to column by a Jasco PU-2080 Plus pump (Tokyo, Japan). Sample solution for the GPC measurement was filtered using 0.2 μm pore size membrane filter. The molecular weight of the polymer was calibrated with poly(2-vinylpyridine) standard samples. The M_n and M_w/M_n value for the polymers were estimated as 9,600 g/mol and 1.26, respectively.

Characterization of polystyrene (PS) particles carrying a PDEA steric colloidal stabilizer (PDEA-PS particles)

Optical microscopy

The particles were observed using an optical microscope (Motic BA200, Shimadzu Co. Ltd.) fitted with camera (Moticam 5 plus, Shimadzu Co. Ltd.).

Particle size analysis

The size of the particles was determined using a laser diffraction particle size analyzer (Malvern Mastersizer 2000) equipped with a small volume sample dispersion unit (Hydro 2000SM; *ca.* 150 mL including flow cell and tubing), a HeNe laser (633 nm) and a solid-state blue laser (466 nm). The stirring rate was adjusted to 2000 rpm. The raw data were analyzed using a Malvern software. The mean particle diameter was taken to be the volume equivalent sphere mean diameter (D_v), which is

mathematically expressed as $D_v = \Sigma D_i^4 N_i / \Sigma D_i^3 N_i$, where D_i is the diameter of individual particles and N_i is the number of particles corresponding to the specific diameter. The resulting data are presented as mean diameter ± standard deviation. The laser diffraction method determined particle size and its distribution by measuring the angular variation in intensity of light scattered as a laser beam passes through a dispersed particulate sample. Large particles scatter light at small angles relative to the laser beam and small particles scatter light at large angles. The angular scattering intensity data is then analyzed to calculate the size of the particles responsible for creating the scattering pattern based on the Mie theory.

Chemical composition

CHN elemental microanalyses were conducted using an Element analyzer 2400II (Perkin Elmer, Yokohama, Japan) at Nagasaki University (Office for Research Initiatives and Development, Nagasaki University, Nagasaki, Japan). The PDEA loading percentage was determined by comparing the nitrogen content of the particles with that of the PDEA homopolymer (N = 0.1 wt% for the PDEA-PS particles and 7.6 wt% for the PDEA homopolymer).

^{1}H NMR spectroscopy

^{1}H NMR spectra were obtained using a JEOL JNM-ECZ 400 MHz NMR. ^{1}H NMR samples solutions were prepared in $CDCl_3$.

Zeta potential

Zeta potentials were calculated from the electrophoretic mobility, measured using a Malvern Zetasizer Nano ZS with an MPT-2 Multi-Purpose Titrator. Measurements

were conducted as a function of pH with diluted dispersions (approximately 0.02 w/v%) by gradually adding an aqueous solution of NaOH, starting from an initial pH of approximately 3. Zeta potentials were averaged over 3 runs at each pH.

Synthesis of PS particles carrying a poly(N-vinyl pyrrolidone) (PNVP) steric colloidal stabilizer

The dispersion polymerization of styrene was performed in the presence of the PNVP steric stabilizer in batch mode at 70 °C using 2,2'-azobisisobutyronitrile (AIBN) initiator [S. Fujii, P. D. Iddon, A. J. Ryan, S. P. Armes, *Langmuir* 2006, 22, 7512.]. The synthetic protocol was as follows. PNVP (nominal molecular weight = 360,000; 2.5 g; 10 wt% based on styrene) was added to isopropanol (250 mL) in a round-bottomed 500 mL flask with a magnetic stirrer bar and stirred vigorously at 70 °C until the PNVP had dissolved completely, followed by degassing with a nitrogen purge. Polymerization commenced after the addition of 0.25 g AIBN dissolved in 25.0 g styrene. The reaction was allowed to proceed for 24 h with continuous stirring at 250 rpm under a nitrogen atmosphere. The PS particles were then purified by repeated centrifugation-redispersion cycles, replacing successive supernatants with deionized water, followed by freeze drying.

Preparation of gas marbles

Air bubbles with controlled volumes were injected below the planar air-water interface covered by PDEA-PS particle raft using the syringe, resulting in the formation of bubbles whose upper surfaces are covered by the particle raft. The bubble was then pushed using a dispensing spoon toward the surrounding particle raft and was rolled over it to cover its whole surface. The PDEA-PS particles

autonomously coat the aqueous bubble and render it non-wetting. (See Figure S5 and Movie S1)

Stability of gas marbles at various relative humidities

The gas marbles (bubble volume, 20 μL) were placed in the enclosed dessicator inside of the relative humidity was controlled by saturated aqueous solution of salts[17b]. The relative humidities were tuned to be 40, 50, 62, 74, 80, and 91% using saturated aqueous solutions of magnesium chloride, potassium carbonate, sodium bromide, sodium chloride, potassium chloride, and potassium sulfate, respectively.

Determination of the mean particle mass per one gas marble

After complete evaporation of the water by drying in air at room temperature for 24 h, the residual mass of PDEA-PS particles was determined gravimetrically using a balance (AB135S Analytical Balance, Mettler Toledo). The accuracy of weight is in the order of 0.01 mg.

Results and Discussion Section

Configurations of PDEA on the particle surface

The area occupied by a PDEA chain at the surface of the PS particle was calculated to be 4.37 nm^2 based on the elemental microanalysis results. The square root of the occupied molecular area (2.09 nm) exceeds the diameter of gyration (1.27 nm) of a PDEA chain (degree of polymerization = 51), indicating the random-walk configurations of PDEA on the particle surface.

Motion of gas marbles

The (near)spherical gas marbles could move on the gas-solid surface (elastic field) in a rotating fashion, when a physical force, such as air blowing or knocking, is applied to them (Movies S2 and S3). Additionally, the direction and distance of the motions could be altered by adjusting the direction and strength of the applied forces. Furthermore, gas marbles could also move on a gas-liquid surface (fluid field) as well as gas-solid surface, by application of physical forces, such as air blowing and knocking (Movies S6 and S7). The gas marble could slide on the planar water surface due to surface flows of the fluids (water and air). Creation of a vertical deflection of the water surface around the gas marble could also induce the motion of gas marbles (Movie S8).

Structure of PDEA-PS particle layer on the gas marble

Particle size could be one of main factors, which realize fabrication of gas marbles with a monolayer structure. Particle aggregates could be observed using naked eyes and stereomicroscope in particle raft in the case of PDEA-PS particles with a diameter of 1.58 ± 0.03 μm, because particle-particle interaction dominates over gravity. In this case, gas marbles stabilized with particle aggregates were formed, because the aggregates adsorbed to the bubble surface. On the other hand, larger particles (> a few tens micrometer) should exist as independent, separated particles rather than aggregates, because gravity dominates over the particle-particle interaction [Y. Asaumi, M. Rey, K. Oyama, N. Vogel, T. Hirai, Y. Nakamura, S. Fujii, *Langmuir* 2020, 36, 13274.]. In this case, monolayer-stabilized GMs might be formed because the particles adsorbed to the gas marble surface individually. The particles which did not get into contact with the air-water interface might fall down from the gas marble

during the formation process. Actually, previous studies by Timounay *et al.* indicated the formation of gas marbles with a monolayer structure of PS particles with diameters of 100 and 250 μm. [Y. Timounay, E. Ou, E. Lorenceau, F. Rouyer, *Soft Matter* 2017, 13, 7717; Y. Timounay, O. Pitois, F. Rouyer, *Phys. Rev. Lett.* 2017, 118, 228001.]

Thickness of PDEA-PS particle layer on the gas marble

From the weight of PDEA-PS particles and the diameter of gas marbles, we estimated that, on average, the gas marble coating comprises 21-39 particles, which corresponds to thicknesses of 34-62 μm.

Motion transfer between gas-solid and gas-liquid interfaces

When gas marbles are placed on a water film prepared on a solid substrate (poly(methyl methacrylate)) followed by the induction of sliding motion by air blow, they can move from the water surface to the solid substrate surface (Movie S9). This indicates that the location of gas marble motion can be transferred from the gas-liquid surface (fluid field) to the gas-solid surface (elastic field) by the kinetic energy generated by the air blow and the potential energy due to the water film thickness. This motion transfer can be reversed: gas marbles can move from a solid substrate to a water surface (running up the meniscus of the water film and moving to the horizontal water surface) if the gas marbles on the air-solid surface are given more kinetic energy than the potential energy given by the height of the water film by air blowing (Movie S9).

PDEA-PS particles after disintegration of gas marble on planar air-water interface

After the disintegration of gas marble on planar air-water interface by addition of acid, all particles (aggregates) eventually dispersed in aqueous phase. The acidic solution should wet non-protonated hydrophobic PDEA on PS particles. Wetting of the PDEA-PS particle aggregates did not occur immediately, but took a certain amount of time. The reason why the wetting proceeded gradually rather than immediately should be due to a pinning effect and realization of meta-stable Cassie-Baxter state: the wetting was pinned on the rough surface of the aggregates [K. Aono, K. Ueno, S. Hamasaki, Y. Sakurai, S. I. Yusa, Y. Nakamura, S. Fujii, *Langmuir* 2022, 38, 7603.].

Table S1. Quantitative surface composition of PS homopolymer, PDEA homopolymer and PDEA-PS particles determined by XPS

	Atom / %		
	C	N	O
PDEA-PS particles	93.3	1.3	5.3
PDAE homopolymer	76.3	7.2	16.6
PS homopolymer	99.4	0.0	0.6

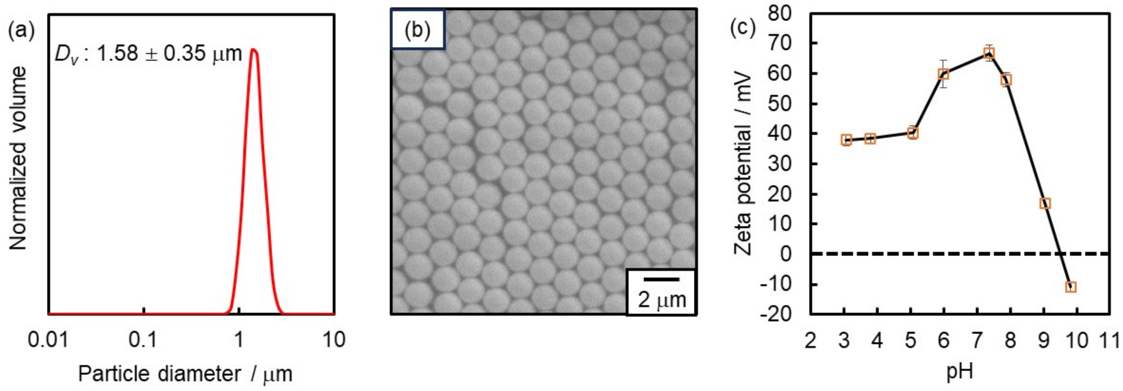

Figure S1. (a) Laser diffraction particle size distribution curve obtained for PDEA-PS particles in isopropanol (IPA), (b) SEM image of the PDEA-PS particles and (c) zeta potentials of the PDEA-PS particles depending on pH for aqueous dispersions.

The volume-average diameter (D_v) in IPA was 1.58±0.35 µm, measured by laser diffraction particle size analysis (Figure S1a). The particle size was confirmed by SEM experiments (Figure S1b). Aqueous electrophoresis studies indicated the existing of PDEA on the surface of PS particles (Figure S1c). The iso-electric point of the PDEA-PS particles was approximately pH 9.5.

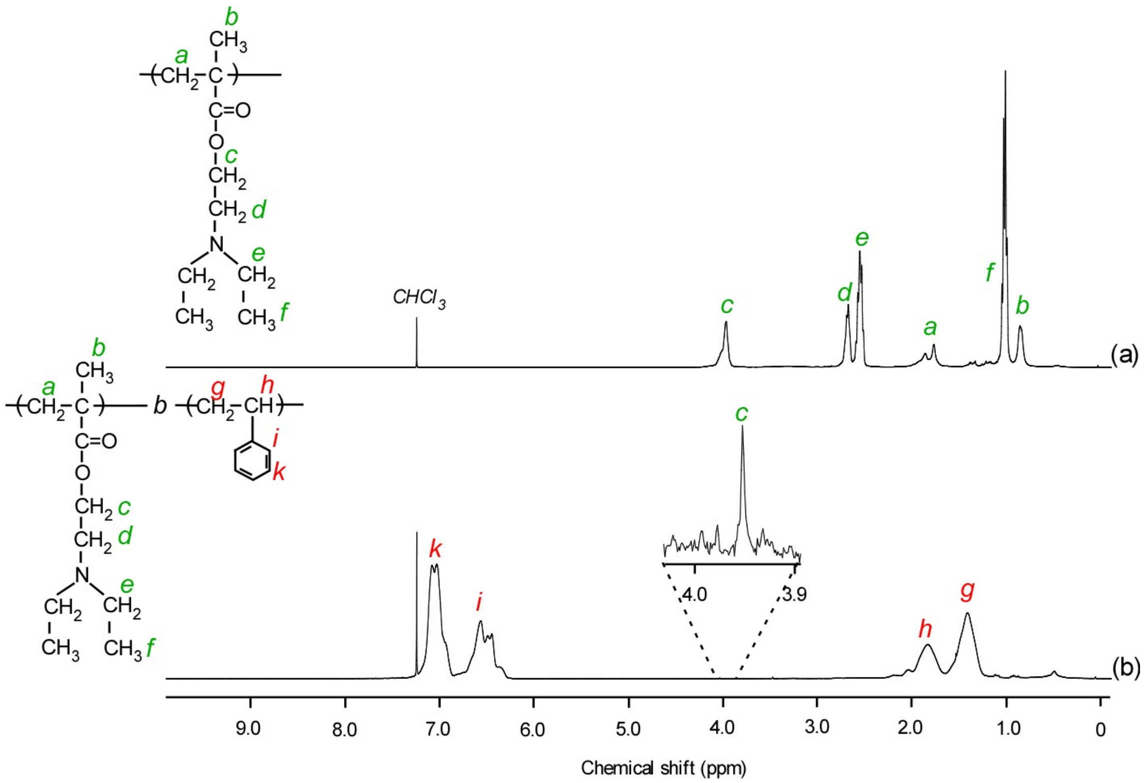

Figure S2. ^{1}H NMR spectra for (a) PDEA and (b) PDEA-PS particles in $CDCl_3$ at room temperature. The resonance band observed at 1.64-2.05 ppm (peak *a*) and 0.87 ppm (peak *b*) were attributed to main chain methylene protons and α-methylene protons, respectively. The resonance band at 1.04 ppm (peak *f*) was associated with the pendant methylene protons from the side chain of the polymer. ^{1}H NMR spectrum for PDEA-PS particles is indicated in Figure S2b. The main chain methylene protons were contributed to the signal bands at 1.23-2.22 ppm (peak *g*, *h*), and the phenyl protons were associated with the resonance bands at 6.40-7.10 ppm (peak *k*, *i*).

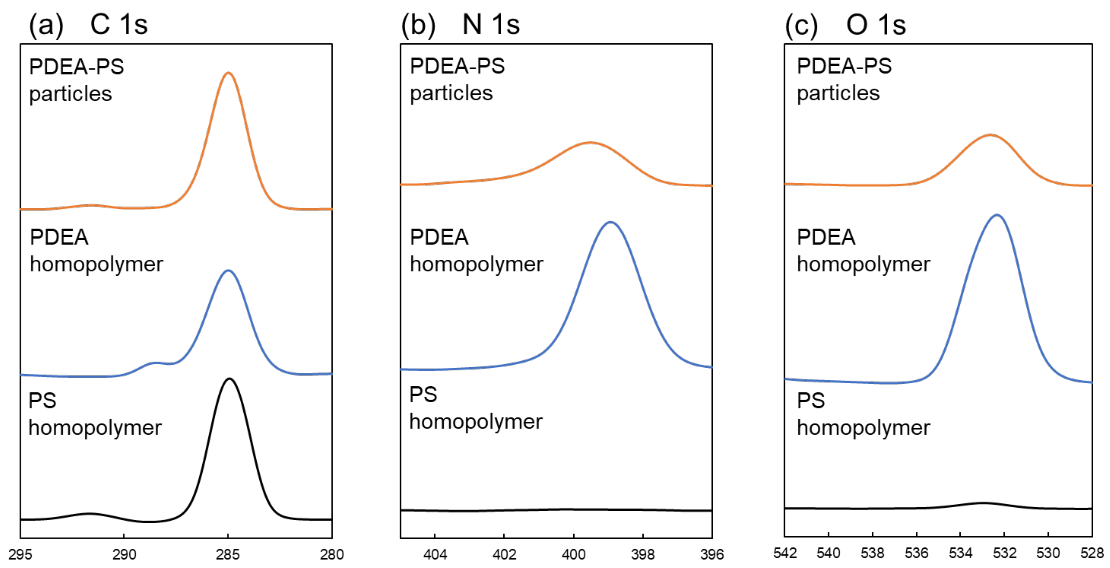

Figure S3. XPS core-line spectra obtained for the PDEA-PS particles, PDEA homopolymer, and PS homopolymer: (a) C 1s, (b) N 1s, and (c) O 1s.

The spectrum of PDEA-PS particles reveals N1s and O1s signals at 399 eV and 532 eV, respectively, which are absent in a reference spectrum recorded for a PS homopolymer. These signals are attributed to one nitrogen atom ($N(CH_2CH_3)_2$) and two oxygen atoms (C=O and C-O) on the methacrylic repeat units of the PDEA stabilizer. Given that the XPS sampling depth is typically only 2-5 nm, these observations provide good evidence that the PDEA stabilizer is present at the surface of the PDEA-PS particles. Moreover, the intensity of the N1s signal obtained for the PDEA-PS particles can be compared to that of the PDEA homopolymer in order to estimate a surface coverage of 18.7 % for the PDEA stabilizer chains on the particle surface.

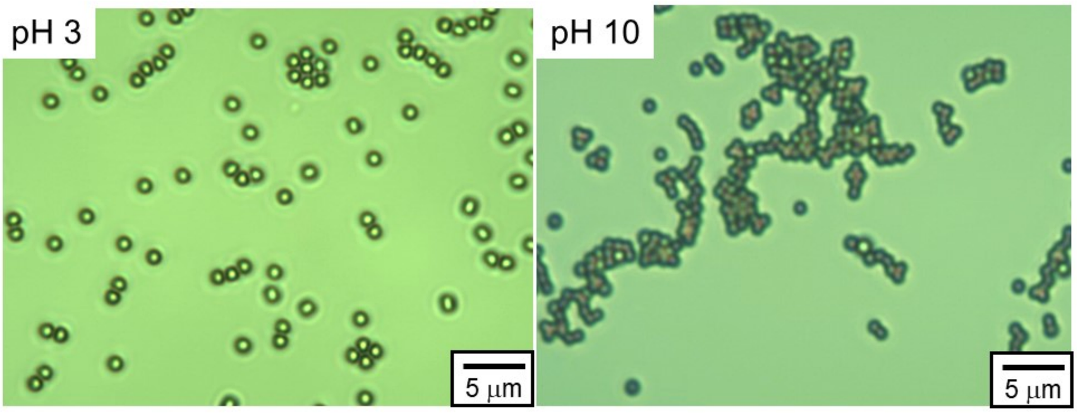

Figure S4. Optical microscopy images of aqueous dispersions of PDEA-PS particles at pH 3 and 10.

Notably, at pH 10, substantial flocculation was observed. This phenomenon occurs because the PDEA stabilizer chains become non-protonated and neutral, rendering them hydrophobic under basic conditions. Conversely, protonation of the PDEA chains at pH 3 results in highly cationic particles with significant electrosteric repulsion, leading to a high degree of dispersion. Consequently, the PDEA-PS particles can exist in two distinct states in aqueous media: (i) as colloidally stable particles with protonated, highly cationic PDEA chains in acidic solutions, and (ii) as flocculated particles with neutral PDEA chains in either neutral or alkaline solutions.

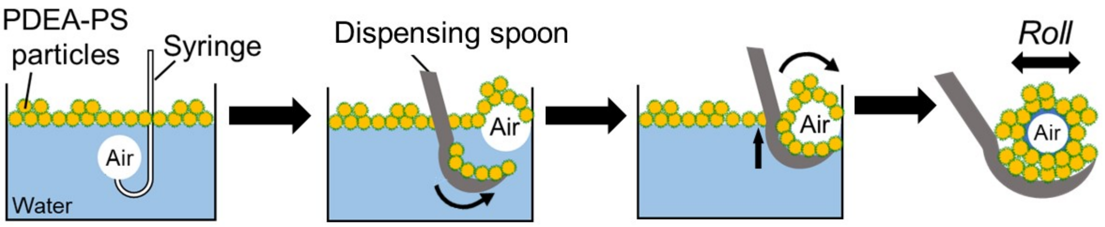

Figure S5. Fabrication method of gas marble. (see also Movie S1)

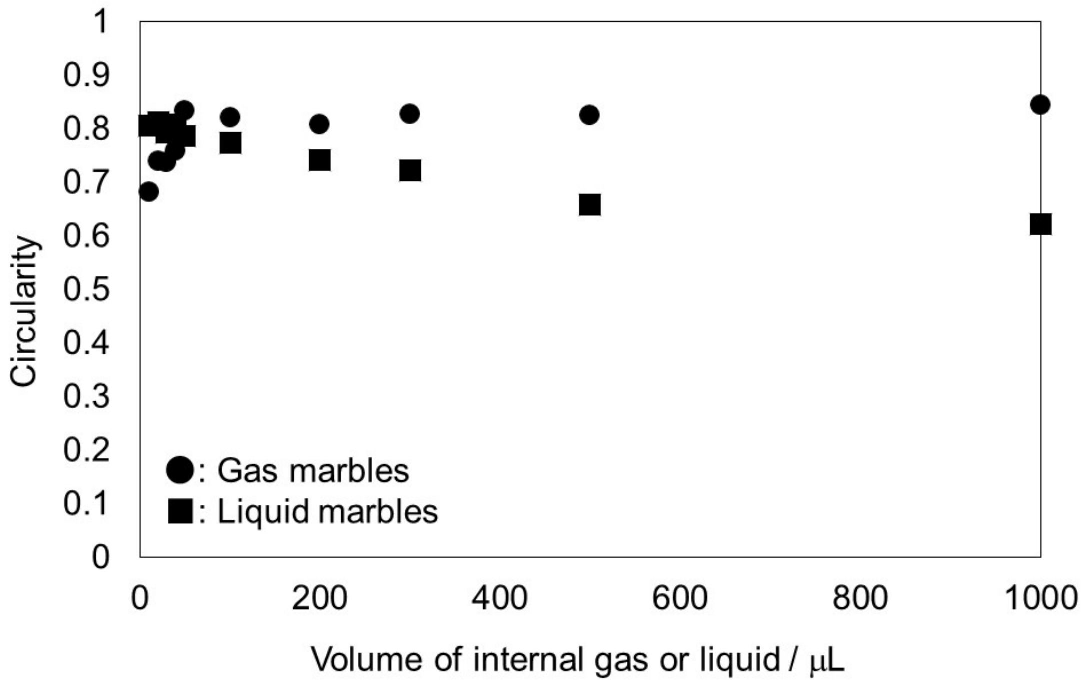

Figure S6. Circularity of gas marbles and liquid marbles depending on volume of internal gas and liquid, respectively.

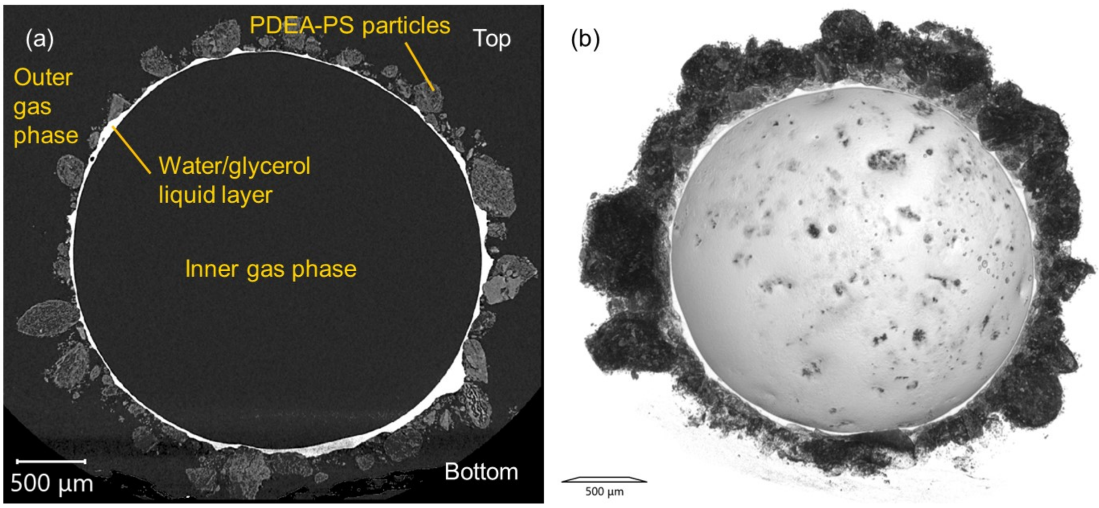

Figure S7. (a) Virtual 2D cross-sectional and (b) virtual 3D X-ray microscopy images illustrating the structure of gas marble (water/glycerol, 1/1, w/w; gas volume, approximately 10 µL)

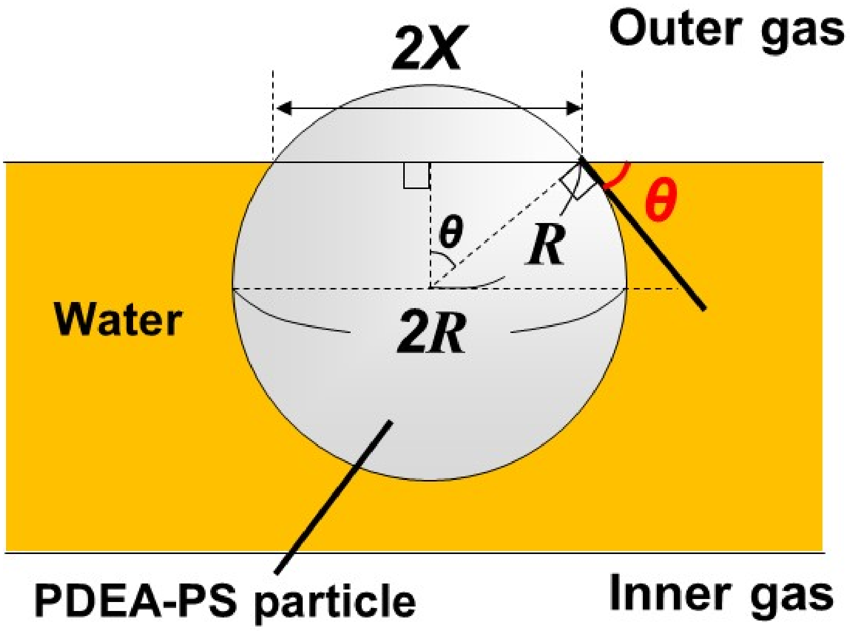

Figure S8. Determination of the contact angle (through water phase) of the PDEA-PS particle at the air-water interface using the SEM images.

The cross-sectional SEM image allows determination of the contact angles (θ) of PSEA-PS particle in the array at air-water interface[17c]. Here, θ is the contact angle measured through the water phase. The contact angles of the particles at the air-water interface was arithmetically determined to be 74 ± 3º.

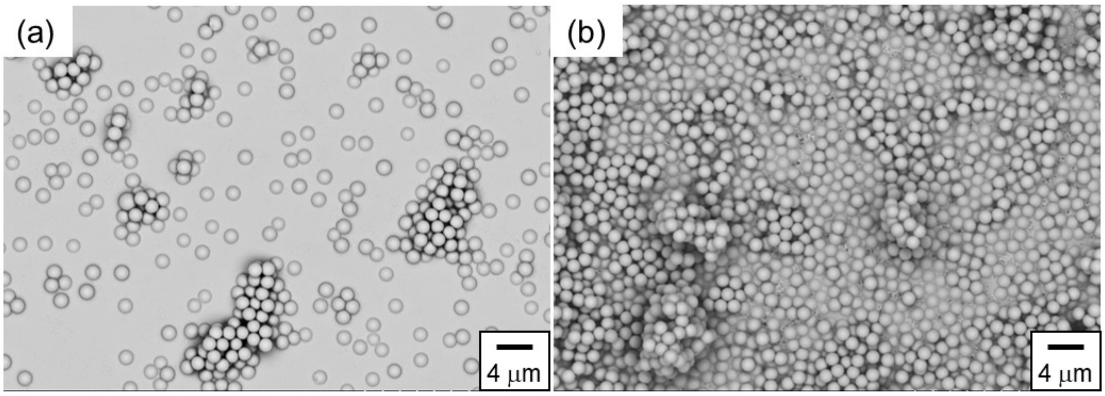

Figure S9. SEM images of planar air-water interface covered by PDEA-PS particle powder after ethyl cyanoacrylate vapor treatment, followed by the evaporation of water. The ethyl cyanoacrylate vapor treatment was conducted (a) before and (b) after application of physical force (knocking the particle powder from the air phase using a plastic spatula). The images were observed from air-phase faced side.

Before the application of physical force, the particle aggregates were mainly found in the PDEA-PS particle raft on planar air-water interface, because particle-particle interaction dominates over gravity for a few micrometer-sized polymer particles. These aggregates should cover the gas marbles. The particle array monolayer was formed after the application of physical force. These results could suggest that the particle array monolayer could be formed during the preparation of gas marbles by rolling, which could break the aggregates partially to the independent PDEA-PS particles.

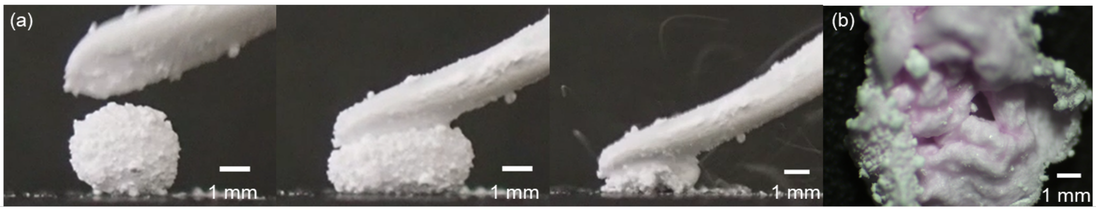

Figure S10. (a) Disruption of gas marble (bubble volume, 20 μL) by mechanical compression. (b) Inner wall of gas marble observed after disruption. Water was dyed using rhodamine B.

List of supporting movies

Supporting Movie S1 Fabrication of gas marble using an air bubble (20 μL).

Supporting Movie S2 Motion of gas marble (air bubble volume, 20 μL) on solid substrate by air blowing.

Supporting Movie S3 Motion of gas marble (air bubble volume, 20 μL) on solid substrate by knocking using a spatula.

Supporting Movie S4 X-ray microscopy movie illustrating the structure of gas marble (water/glycerol, 1/1, w/w; gas volume, approximately 10 μL).

Supporting Movie S5 Disruption of gas marble (just after fabrication, air bubble volume, 20 μL) by application of mechanical stress using a spatula.

Supporting Movie S6 Motion of gas marble (air bubble volume, 20 μL) on planar air-water interface by air blowing.

Supporting Movie S7 Motion of gas marble (air bubble volume, 20 μL) on planar air-water interface by knocking using a spatula.

Supporting Movie S8 Motion of gas marble (air bubble volume, 20 μL) on planar air-water interface by creation of a vertical deflection around the gas marble using a hydrophobic spatula.

Supporting Movie S9 Amphibious motion of gas marble (air bubble volume, 20 μL).

Supporting Movie S10 Disruption of gas marble (air bubble volume, 20 μL) on planar air-water interface by addition of aqueous solution of HCl to the bulk aqueous subphase.

Supporting Movie S11 Disruption of gas marble (air bubble volume, 20 μL) on planar air-water interface by UV irradiation to the bulk aqueous subphase containing a photoacid generator, diphenyliodonium nitrate.
